# Causal relationships between height and weight with distal tibia microarchitecture and geometry in adult female twin pairs

**DOI:** 10.1093/jbmrpl/ziae095

**Published:** 2024-07-18

**Authors:** Frida Igland Nissen, Vivienne F C Esser, Åshild Bjørnerem, Ann Kristin Hansen

**Affiliations:** Department of Clinical Medicine, UiT The Arctic University of Norway, Hansine Hansens veg 18, 9019 Tromsø, Norway; Department of Orthopedic Surgery, University Hospital of North Norway, Hansine Hansens veg 67, 9019 Tromsø Norway; Department of Obstetrics and Gynecology, University Hospital of North Norway, Hansine Hansens veg 67, 9019 Tromsø, Norway; Centre for Epidemiology and Biostatistics, Melbourne School of Population and Global Health, University of Melbourne, 207 Bouverie St, Carlton VIC 3053, Australia; Department of Clinical Medicine, UiT The Arctic University of Norway, Hansine Hansens veg 18, 9019 Tromsø, Norway; Department of Obstetrics and Gynecology, University Hospital of North Norway, Hansine Hansens veg 67, 9019 Tromsø, Norway; Norwegian Research Centre for Women’s Health, Oslo, University Hospital, Sognsvannsveien 20, 0372 Oslo, Norway; Department of Clinical Medicine, UiT The Arctic University of Norway, Hansine Hansens veg 18, 9019 Tromsø, Norway; Department of Orthopedic Surgery, University Hospital of North Norway, Hansine Hansens veg 67, 9019 Tromsø Norway

**Keywords:** bone structure, height, high-resolution peripheral quantitative computed tomography, ICE FALCON, osteoporosis, weight

## Abstract

Higher stature and lower weight are associated with increased risk of fracture. However, the pathophysiology for the associations of height and weight with bone microarchitecture and geometry is unclear. We examined whether these associations were consistent with causation and/or with shared familial factors. In this cross-sectional study of 566 female twins aged 26-76 yr, a regression analysis for twin data, Inference about Causation by Examination of FAmilial CONfounding (ICE FALCON), was used for testing causation. The bone microarchitecture and geometry of the distal tibia was assessed using HR-pQCT and the StrAx1.0 software. Higher stature was associated with larger total bone cross-sectional area (CSA), lower total bone volumetric bone mineral density (vBMD), larger cortical CSA, thinner cortices, higher porosity of the total cortex, compact cortex, outer and inner transitional zone (TZ), lower cortical vBMD, and larger medullary CSA (regression coefficients (*β*) ranging from −.37 to .60, all *p*<.05). Using ICE FALCON, the cross-pair cross-trait associations attenuated toward zero after adjusting for the within-individual association (absolute values of *β* ranging from .05 to .31, all *p*<.001). Higher weight was associated with higher total bone vBMD, larger cortical CSA and thicker cortices, lower porosity of the total cortex and inner TZ, and higher cortical vBMD (*β* ranging from −.23 to .34, all *p*<.001), and thinner trabeculae, higher trabecular number, lower trabecular separation, and higher trabecular vBMD (*β* ranging from −.31 to .39, all *p*<.05). Only cortical CSA attenuated toward zero after adjusting for the within-individual association between weight and bone microarchitecture (β = .042, *p*=.046). Higher stature was associated with a weaker cortical, not trabecular bone traits, whereas higher weight was associated with stronger cortical and trabecular bone traits. The results were consistent with height having a causal effect on weaker cortical bone structure, whereas weight had a casual effect on the larger cortical CSA.

## Graphical Abstract

**Graphical Abstract:**
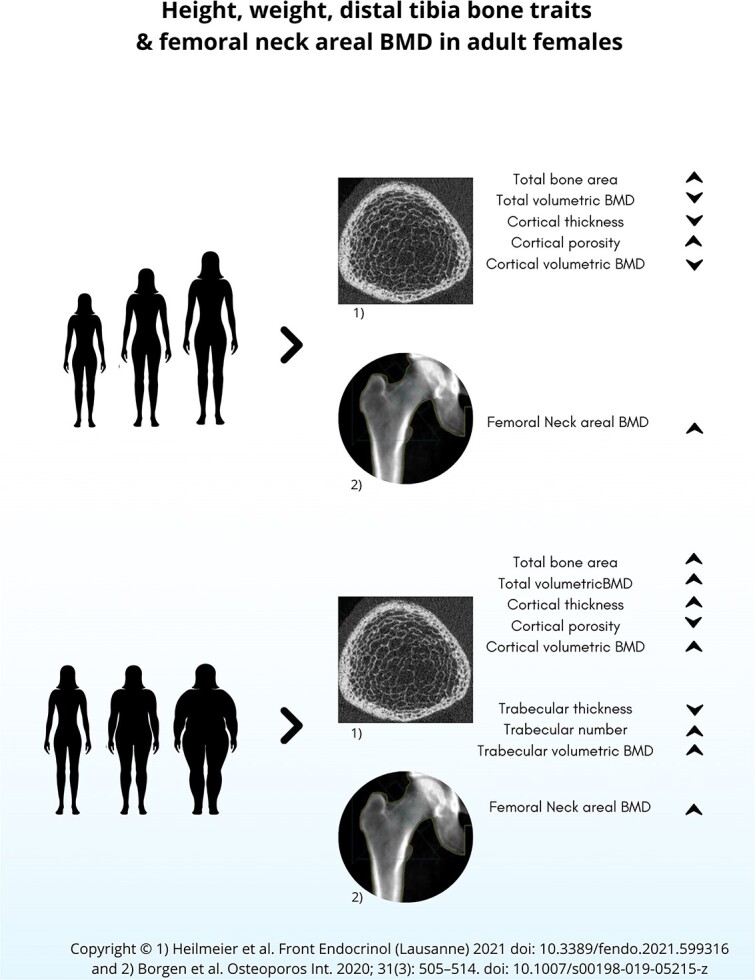

## Introduction

Taller women have an increased risk of hip, wrist, and vertebral fractures.^1-6^ A plausible explanation is the greater impact on landing resulting from falling from a greater height. A large prospective cohort study of 796 081 postmenopausal women in the UK with 8 yr follow-up, showed that the risk of fracturing the femoral neck (FN) increased with 48% per 10-cm increase in height.^1^ Furthermore, the overall risk of fracture at nine different sites (humerus, radius and/or ulna, wrist, femur, neck of femur, patella, tibia, and/or fibula, ankle, clavicle/spine/rib) increased with 21% per 10-cm increase in height. Yet, a global multicenter cohort study of 52 939 postmenopausal women showed that shorter women had more fractures of the clavicle and upper arm.^2^

The individual height is established during growth following physeal closure. It has been suggested that bone resorption stimulate periosteal expansion in a study that reported C-terminal cross-linking telopeptide of type I collagen (CTX) associated with periosteal circumference at the distal tibia of adolescents.^7^ During 3 yr follow-up, little bone loss was seen in women who remained premenopausal (mean age 40.7 yr), the largest bone loss was observed in women who transitioned from the perimenopausal to postmenopausal stage (mean age 51.8 yr), and 80% of the bone loss at the distal tibia across menopause was cortical.^8^ Intracortical remodeling is reported associated with larger bone size and suggested to result from a local regulation of remodeling that is suppressed in slender bones, but not in larger bones.^9^ A cross-sectional twin study of 345 women aged 40-61 yr showed that taller women had wider bones, a relatively wider medullary canal and relatively thinner cortices at the distal tibia, which could explain that longer bones are more prone to fracture.^10^ However, this study did not investigate whether height was associated with porosity of the cortical compartments or trabecular bone microarchitecture. Another study showed that taller postmenopausal women had wider bones at the proximal femur, and higher stature was associated with increased bone turnover marker (CTX).^11^ Bone turnover markers are associated with larger intracortical surface area and smaller trabecular surface area at the distal tibia in women aged 40-61 yr.^12^ To the best of our knowledge, it is not known whether height is associated with trabecular bone traits, and the reasons for the association between height and bone traits are not fully understood.

Weight is another risk factor for fracture, and similarly to height, the effect is divergent; lower weight is associated increased risk of hip, spine, and wrist fractures,^2^^,^^13-15^ whereas higher weight or BMI is associated with increased risk of ankle, lower leg, and humerus fractures and decreased risk of hip fractures.^2^^,^^14-17^ Higher BMI is associated with increased BMD.^18-20^ However, increased body fat is shown to introduce errors in areal BMD (aBMD) measurements by DXA,^21^^,^^22^ and the higher aBMD values with higher weight might be due to this artifact. Bone measurements by using high-resolution peripheral quantitative computed tomography (HR-pQCT) are less affected by overlying fat than using DXA.^21^

A cross-sectional study using HR-pQCT of the distal radius and distal tibia of 491 women showed that a higher BMI category significantly increased cortical area, cortical thickness, and cortical volumetric BMD (vBMD), as well as increased trabecular number, trabecular vBMD, and a decreased cortical porosity, trabecular thickness, and trabecular separation.^23^ Similarly, a cross-sectional study of 63 obese and 126 normal-weight postmenopausal women showed higher total, cortical, and trabecular vBMD, larger cortical area, lower cortical porosity, and higher trabecular number at the distal tibia in obese women.^24^ However, the increase in absolute values of the bone traits was not in proportion to the excess of weight or BMI, suggesting a relative bone fragility.^23^^,^^24^ In a nested case–control study of women 50 yr or older, higher weight was associated with larger bone size, and thicker cortices of the proximal femur as well as lower levels of bone resorption (CTX).^11^ This suggests that a lower bone turnover may result from estrogen produced in increasing amounts of fat tissue. Higher aBMD and stronger bone structure by higher weight may also reflect the bone’s adaptation to increased load. However, it is unclear whether this adaptation results in greater bone strength, thus providing protection of fractures.

The associations between height, weight, and bone traits can be mediated by different mechanisms and influenced by confounders. However, associations as such can be unspecific without knowing the direction or causes behind the associations. Showing evidence for a causal component gives associations both strength and directions. Performing randomized studies with long follow-up can be costly. For these reasons, there is an increasing interest to use statistical methods that provides evidence for causal effects in cross-sectional data. One such method is “The Inference about Causation by Examination of FAmilial CONfounding” (ICE FALCON), in which familial confounding between the variables is examined.^25^ We hypothesized that height and weight are independently associated with distal tibial microarchitecture in women, and that height and weight influence the bone traits differently, ie, height is mainly associated with cortical bone traits, because intracortical remodeling is associated with larger bone size, whereas weight is associated with both cortical and trabecular bone traits.^20^^,^^23^^,^^24^^,^^26^ In addition to exploring associations, we aimed to go one step further by examining whether the relationships of height and weight with bone microarchitecture are consistent with causation.

## Materials and Methods

### Subjects

A cross-sectional study was conducted in Melbourne, Australia, in 2008-2011, and included 605 female twins (369 monozygotic (MZ) and 236 dizygotic (DZ)) aged 26-76 yr.^10^^,^^12^^,^^27^^,^^28^ Women who were currently using menopausal hormone therapy, those with movement artifacts on the HR-pQCT scans, and those without data for their twin were excluded. For the final analysis, 566 twins (344 MZ and 222 DZ) aged 26-76 yr were included. All participants provided written informed consent, and the study was approved by The Austin Health Human Research Ethics Committee (H2008/03151).

### Bone microarchitecture and other measurements

HR-pQCT (XtremeCT, Scanco Medical AG, Brüttisellen, Switzerland, isotropic resolution of 82 μm) was used to obtain images of the distal tibia.^29^^,^^30^ The scanning was performed at the same side as the nondominant hand. The opposite side was scanned if the participant had a fracture or osteosynthesis material at the nondominant tibia. The region of interest (ROI) included 110 CT slices obtained at a standardized distance of 22.5 mm from a reference line at the endplate of the distal tibia. The proximal 49 slices were chosen because the cortices are thicker in this region. The StrAx1.0 software was used to quantify the following bone traits: total bone cross-sectional area (CSA), total vBMD, cortical CSA, cortical thickness, porosity of the total cortex, compact cortex, outer- and inner transitional zone (TZ), cortical vBMD, medullary CSA, trabecular thickness, trabecular number, trabecular separation, and trabecular vBMD.^31^ In contrast to the threshold-based software Scanco that quantifies porosity of the compact cortex, StrAx1.0 is a density-based method that also includes the transitional zone. StrAx1.0 automatically selects the attenuation profile curves with two plateaus corresponding to the compact-appearing cortex and the trabecular compartment. The transitional zone is represented by the S-shaped curve between these two plateaus. StrAx1.0 quantifies porosity as a fraction of void regardless of the size of the pores, and accounts for the partial volume effect by including partly empty voxels resulting in higher values of porosity when using the StrAx1.0 method compared with Scanco.^31^ FN, total hip, and spine aBMD was quantified using DXA (Lunar, Madison, WI, USA). Height and weight were measured without shoes and with light clothing.

### Statistical methods

The outcome variables were the standardized residuals of the bone traits after adjustment for covariates using a semi-parametric model to account for potential nonlinear relationships. Analyses of the associations of bone traits with height were adjusted for age and weight. Conversely, the associations of bone traits with weight were adjusted for age and height. When considered as predictive variables, height and weight were both standardized, with weight also log-transformed to achieve approximate normality. All analyses were performed using generalized estimating equations (GEEs) to account for the correlation within twins in a pair.^32^

We firstly considered whether there was evidence for a difference in predictive variables, covariates, and unadjusted bone traits between MZ and DZ twins. Second, the within-individual associations between each predictive variable and bone traits were assessed. Third, bone traits with significant within-individual associations were further assessed using the ICE FALCON method.^25^ In brief, the within-individual association and cross-pair cross-trait association (the association between the predictor in an individual’s twin and the outcome in the individual) were compared when assessed separately and together. If, when assessed together, the cross-pair cross-trait association attenuates toward zero, while the within-individual association remains unchanged, the results are consistent with causation from predictor to outcome. If both within-individual and cross-pair cross-trait association attenuate to the same degree, the results are consistent with confounding from shared familial factors (ie, genetic or shared environmental), further referred to as familial confounders. If a mixture of these two scenarios is observed, the results are consistent with both causation and familial confounding.^32^ The analyses were conducted using the R (https://www.R-project.org/) package «geepack». Residuals of the bone traits were found using the R package «SemiPar».^33^

## Results

The mean age of the 566 female twins was 48 yr (SD 10.8). Height, weight, and bone traits were similar between zygosities (Table 1).

**Table 1:** Characteristics of the participants and distal tibia bone traits, aBMD of the FN, total hip and spine, and comparison of DZ and MZ twins using GEEs.

	All women (*n* = 566)		DZ (*n* = 222)		MZ (*n* = 344)		
**Characteristics**	Mean	SD	Mean	SD	Mean	SD	*p*
**Age (yr)**	48.1	10.8	48.4	10.3	47.9	11.1	.69
**Height (cm)**	163	6.46	164	6.55	163	6.36	.07
**Weight (kg)**	70.2	15.5	70.6	16.3	70.0	15.0	.70
**Distal tibia bone traits**							
**Total bone CSA (mm^2^)**	623	102	632	102	618	101	.23
**Total vBMD (mg HA/cm^3^)**	310	57.1	310	57.4	310	57.1	.98
**Cortical CSA (mm^2^)**	204	22.2	205	21.2	203	22.8	.57
**Cortical thickness (mm)**	2.35	0.25	2.34	0.25	2.35	0.26	.55
**Total cortex porosity (%)**	60.2	6.16	60.4	6.10	60.1	6.19	.64
**Compact cortex porosity (%)**	42.0	7.21	42.0	7.09	42.0	7.30	.95
**Outer TZ porosity (%)**	43.0	6.36	43.1	6.42	42.9	6.33	.84
**Inner TZ porosity (%)**	84.9	3.05	84.6	3.11	85.0	3.00	.26
**Cortical vBMD (mg HA/cm^3^)**	662	77.6	660	77.1	664	78.0	.64
**Medullary CSA (mm^2^)**	419	92.0	427	93.4	414	90.8	.23
**Trabecular thickness (mm)**	0.19	0.01	0.19	0.01	0.19	0.01	.56
**Trabecular number (1/mm)**	2.69	0.63	2.69	0.67	2.68	0.61	.85
**Trabecular separation (mm)**	1.35	0.29	1.33	0.30	1.36	0.29	.33
**Trabecular vBMD (mg HA/cm^3^)**	131	39.3	134.9	40.6	129	38.3	.18
**Femoral Neck aBMD (g/cm^2^)**	0.97	0.14	0.98	0.14	0.96	0.15	.18
**Total Hip aBMD (g/cm^2^)**	1.00	0.15	1.01	0.14	0.99	0.15	.28
**Spine aBMD (g/cm^2^)**	1.19	0.18	1.19	0.17	1.18	0.18	.45

Abbreviations: CSA = cross-sectional area; vBMD = volumetric bone mineral density; HA = hydroxyapatite; TZ = transitional zone; aBMD = areal bone mineral density; monozygotic = MZ; dizygotic = DZ; Femoral neck = FN; generalized estimating equation = GEE. The sample size for some variables is lower than listed due to missing values.

Associations of height and weight with distal tibia bone traits are presented in Table 2. Higher stature was associated with larger total and cortical CSA, thinner cortices, higher porosity of the total cortex, compact cortex, and outer and inner TZ. Of bone traits in the trabecular compartment, higher stature was associated with larger medullary CSA. Higher stature was associated with lower total and cortical vBMD, and higher FN aBMD.

**Table 2:** Within-individual associations between height and weight and distal tibia bone traits and aBMD of the FN, total hip, and spine.

	**Height**			**Weight**		
**Bone traits**	**Coef**	**SE**	***p***	**Coef**	**SE**	***p***
**Total bone CSA (mm^2^)**	0.600	0.042	**<.001**	0.102	0.051	**.046**
**Total vBMD (mg HA/cm^3^)**	−0.341	0.046	**<.001**	0.278	0.043	**<.001**
**Cortical CSA (mm^2^)**	0.228	0.050	**<.001**	0.341	0.043	**<.001**
**Cortical thickness (mm)**	−0.278	0.046	**<.001**	0.289	0.045	**<.001**
**Total cortex porosity (%)**	0.372	0.042	**<.001**	−0.210	0.044	**<.001**
**Compact cortex porosity (%)**	0.375	0.043	**<.001**	−0.078	0.048	.105
**Outer TZ porosity (%)**	0.257	0.044	**<.001**	−0.047	0.046	.303
**Inner TZ porosity (%)**	0.142	0.049	**.004**	−0.232	0.045	**<.001**
**Cortical vBMD (mg HA/cm^3^)**	−0.365	0.042	**<.001**	0.220	0.043	**<.001**
**Medullary CSA (mm^2^)**	0.597	0.042	**<.001**	0.008	0.053	.885
**Trabecular thickness (mm)**	0.051	0.049	.299	−0.114	0.044	**.009**
**Trabecular number (1/mm)**	0.019	0.046	.683	0.387	0.040	**<.001**
**Trabecular separation (mm)**	−0.059	0.049	.236	−0.310	0.043	**<.001**
**Trabecular vBMD (mg HA/cm^3^)**	0.058	0.049	.239	0.232	0.046	**<.001**
**Femoral Neck aBMD (g/cm^2^)**	0.168	0.047	**<.001**	0.336	0.042	**<.001**
**Total Hip aBMD (g/cm^2^)**	0.010	0.050	.836	0.431	0.040	**<.001**
**Spine aBMD (g/cm^2^)**	0.070	0.051	.163	0.231	0.046	**<.001**

Abbreviations: Coef = regression coefficient; SE = standard error; CSA = cross-sectional area; vBMD = volumetric bone mineral density; HA = hydroxyapatite; TZ = transitional zone; aBMD = areal bone mineral density; Femoral neck = FN.

Higher weight was associated with larger total and cortical CSA, thicker cortices, and lower porosity of the total cortex and the inner TZ. In contrast to height, higher weight was associated with thinner trabeculae, higher trabecular number, and lower trabecular separation. Higher weight was associated with higher total, cortical, and trabecular vBMD, as well as higher FN, total hip, and spine aBMD.

ICE FALCON analyses were conducted of the bone traits that were significantly associated with height or weight in Table 2. Significant cross-pair cross-trait associations of height with total bone CSA, total vBMD, cortical CSA, cortical thickness, total cortex and compact cortex porosity, OTZ porosity, cortical vBMD, medullary CSA, and FN aBMD were identified (Table 3). Following adjustment for the within-individual association, all these cross-pair cross-trait associations attenuated to zero and the changes from before and after adjustment were significant (absolute value of regression coefficients (β) ranging from .05 to .31, all *p*<.001, except for FN aBMD, which had a *p*=.014). The results indicate that height has a causal effect on distal tibia cortical microarchitecture, and total, cortical, and medullary CSA, total and cortical vBMD, and FN aBMD, with no evidence for familial confounding. Weight in an individual’s twin was associated with cortical CSA in the individual (Table 4). The cross-pair cross-trait association of weight and cortical CSA did not remain significant after adjustment for the within-individual association and the change from before and after adjustment was significant (β = .042, *p*=.046), suggesting that weight has a casual effect on cortical CSA. None of the other cross-pair cross-trait associations with weight were significant.

**Table 3:** ICE FALCON analyses for associations of height with distal tibia bone traits and femoral neck aBMD.

		**Model I**			**Model II**			**Model III**			**Change** ^**a**^		
		**Coef**	**SE**	***p***	**Coef**	**SE**	***p***	**Coef**	**SE**	***p***	**Coef**	**SE**	***p***
**Total bone CSA (mm^2^)**		0.600	0.042	**<.001**				0.610	0.047	**<.001**	−0.010	0.017	.541
					0.284	0.050	**<.001**	−0.028	0.045	.535	0.312	0.043	**<.001**
**Total vBMD (mg HA/cm^3^)**		−0.341	0.046	**<.001**				−0.338	0.052	**<.001**	−0.003	0.019	.870
					−0.135	0.051	**.008**	−0.009	0.054	.870	−0.126	0.030	**<.001**
**Cortical CSA (mm^2^)**		0.228	0.050	**<.001**				0.238	0.060	**<.001**	−0.010	0.030	.738
					0.104	0.052	**.046**	−0.020	0.060	.739	0.124	0.032	**<.001**
**Cortical thickness (mm)**		−0.278	0.046	**<.001**				−0.275	0.058	**<.001**	−0.003	0.026	.901
					−0.137	0.046	**.003**	−0.007	0.058	.902	−0.130	0.034	**<.001**
**Total cortex porosity (%)**		0.372	0.042	**<.001**				0.375	0.051	**<.001**	−0.003	0.024	.899
					0.170	0.048	**<.001**	−0.007	0.055	.899	0.177	0.034	**<.001**
**Compact cortex porosity (%)**		0.375	0.043	**<.001**				0.399	0.052	**<.001**	−0.024	0.025	.346
					0.151	0.047	**.001**	−0.051	0.053	.336	0.202	0.036	**<.001**
**Outer TZ porosity (%)**		0.257	0.044	**<.001**				0.263	0.050	**<.001**	−0.006	0.021	.775
					0.098	0.048	**.040**	−0.015	0.052	.774	0.113	0.027	**<.001**
**Inner TZ porosity (%)**		0.142	0.049	**.004**				0.135	0.056	**.015**	0.006	0.022	.770
					0.069	0.051	.178	0.017	0.058	.771	0.052	0.024	**.028**
**Cortical vBMD (mg HA/cm^3^)**		−0.365	0.042	**<.001**				−0.365	0.051	**<.001**	0.000	0.024	.986
					−0.171	0.048	**<.001**	0.001	0.055	.986	−0.172	0.033	**<.001**
**Medullary CSA (mm^2^)**		0.597	0.042	**<.001**				0.605	0.047	**<.001**	−0.008	0.015	.615
					0.267	0.051	**<.001**	−0.023	0.046	.609	0.291	0.044	**<.001**
**Femoral Neck aBMD (g/cm^2^)**		0.168	0.047	**<.001**				0.148	0.053	**.005**	0.021	0.021	.319
					0.111	0.052	**.033**	0.058	0.058	.315	0.053	0.021	**.014**

Abbreviations: ICE FALCON = Inference about Causation through Examination of FAmiliaL CONfounding; Coef = regression coefficient; CSA = cross-sectional area; vBMD = volumetric bone mineral density; HA = hydroxyapatite; TZ = transitional zone; aBMD = areal bone mineral density.

Model I included within-individual associations, Model II included cross-pair cross-trait association, and Model III included both.

The analyses of the associations of height with bone traits were adjusted for age and weight. All *p*-values are two-sided.

^a^Changes in regression coefficients of the cross-pair cross-trait association from before and after adjustment for the within-individual association.

**Table 4:** ICE FALCON analyses for associations of weight with distal tibia bone traits and aBMD of the hip and spine.

		**Model I**			**Model II**			**Model III**			**Change** ^**a**^		
		**Coef**	**SE**	***p***	**Coef**	**SE**	***p***	**Coef**	**SE**	***p***	**Coef**	**SE**	***p***
**Total bone CSA (mm^2^)**		0.102	0.051	**.046**				0.105	0.050	**.036**	−0.003	0.005	.540
					0.047	0.048	.320	0.053	0.047	.254	−0.006	0.008	.467
**Total vBMD (mg HA/cm^3^)**		0.278	0.043	**<.001**				0.278	0.043	**<.001**	−0.000	0.001	.866
					0.007	0.047	.880	0.012	0.041	.775	−0.005	0.021	.826
**Cortical CSA (mm^2^)**		0.341	0.043	**<.001**				0.334	0.043	**<.001**	0.007	0.007	.300
					0.105	0.049	**.032**	0.052	0.043	.217	0.042	0.026	**.046**
**Cortical thickness (mm)**		0.289	0.045	**<.001**				0.288	0.045	**<.001**	0.001	0.005	.809
					0.044	0.050	.381	0.011	0.044	.804	0.033	0.023	.152
**Total cortex porosity (%)**		−0.210	0.044	**<.001**				−0.212	0.044	**<.001**	0.002	0.004	.547
					0.007	0.046	.881	0.026	0.041	.533	−0.019	0.016	.232
**Inner TZ porosity (%)**		−0.232	0.045	**<.001**				−0.232	0.044	**<.001**	−0.000	0.003	.972
					−0.035	0.043	.411	−0.035	0.041	.394	−0.000	0.017	.970
**Cortical vBMD (mg HA/cm^3^)**		0.220	0.043	**<.001**				0.222	0.044	**<.001**	−0.002	0.004	.579
					−0.003	0.046	.943	−0.023	0.040	.568	0.020	0.016	.229
**Trabecular thickness (mm)**		−0.114	0.044	**.009**				−0.113	0.043	**.009**	−0.001	0.003	.738
					0.023	0.044	.595	0.016	0.042	.705	0.007	0.009	.420
**Trabecular number (1/mm)**		0.387	0.040	**<.001**				0.393	0.039	**<.001**	−0.006	0.006	.328
					−0.021	0.044	.630	0.044	0.036	.220	−0.066	0.029	**.023**
**Trabecular separation (mm)**		−0.310	0.043	**<.001**				−0.311	0.042	**<.001**	0.001	0.004	.701
					−0.036	0.042	.391	−0.047	0.039	.219	0.011	0.023	.633
**Trabecular vBMD (mg HA/cm^3^)**		0.232	0.046	**<.001**				0.236	0.044	**<.001**	−0.004	0.006	.549
					0.062	0.041	.137	0.074	0.039	.061	−0.012	0.017	.480
**Femoral Neck aBMD (g/cm^2^)**		0.336	0.042	**<.001**				0.336	0.042	**<.001**	−0.000	0.001	.892
					0.005	0.046	.914	0.009	0.035	.794	−0.004	0.025	.868
**Total Hip aBMD (g/cm^2^)**		0.431	0.040	**<.001**				0.434	0.040	**<.001**	−0.003	0.004	.449
					−0.008	0.049	.875	0.036	0.034	.295	−0.044	0.032	.177
**Spine aBMD (g/cm^2^)**		0.231	0.046	**<.001**				0.241	0.043	**<.001**	−0.010	0.009	.316
					−0.019	0.045	.680	0.041	0.039	.287	−0.060	0.020	**.003**

Abbreviations: ICE FALCON = Inference about Causation through Examination of FAmiliaL CONfounding; Coef = regression coefficient; CSA = cross-sectional area; vBMD = volumetric bone mineral density; HA = hydroxyapatite; TZ = transitional zone; aBMD = areal bone mineral density. Model I included within-individual associations, Model II included cross-pair cross-trait association, and Model III included both. The bone traits associations were adjusted for age and height. All *p*-values are two-sided.

^a^Changes in regression coefficients of the cross-pair cross-trait association from before and after adjustment for the within-individual association.

## Discussion

For women aged 26-76 yr, higher stature was associated with a weaker cortical microarchitecture of the distal tibia. Taller women had thinner cortices, higher cortical porosity, lower total, and cortical vBMD. In contrast, higher weight was associated with improved cortical and trabecular bone traits. Following the reasoning of the ICE FALCON approach, the results were consistent with height having a causal effect on bone traits, without familial confounding. Adult height is determined by both genetic and environmental factors. The absence of familial confounding in our results indicates that there are different genes or environmental factors causing height and bone traits. Accordingly, these findings were consistent with height itself having a causal effect on the bone microarchitecture. The results were consistent with weight having a causal effect on cortical CSA, but none of the other bone traits.

This study confirmed the results from a cross-sectional study including 345 women aged 40-61 yr, showing that taller women had wider bones, thinner cortices relative to its CSA, and more porous cortices within each of the cortical compartments.^10^ We additionally showed that height was associated with thinner cortices in absolute values and tested consistency with causation using the ICE FALCON analysis. Furthermore, we included results from DXA-scans, which enabled comparison of 2D aBMD with 3D vBMD. Taller women assemble a relatively lighter skeleton, and the cortical and trabecular compartments are emptier compared with shorter women.^10^ This is due to larger medullary cavities without differences in trabecular microarchitecture, and increased porosity of the compact cortex, the outer and inner transitional zone, and thinner cortices.

Higher stature was associated with increased FN aBMD, which gives the impression of a higher density of the bone in taller individuals. However, the 2D images provided by DXA scans do not consider the width of the bones. As taller women have wider bones, with larger medullary cavities and thinner cortices, the aBMD will be falsely high. It is important for clinicians to be aware of this when evaluating DXA scans and diagnosing osteoporosis, because taller women do not have more “dense” bone. In fact, they have lower vBMD with a lower “true density.” Still, the 2D aBMD works well as a predictor of fracture risk as the falsely high aBMD in taller individuals is due to a larger bone size.^34^ In a larger bone, the bone strength is increased because the cortices are spaced further from the long axis of the bone.^35^

Although most studies on weight or BMI and fracture risk have reported a lower overall risk of fracture with higher weight,^13^^,^^14^^,^^36-39^ the risk of fracturing the ankle, upper leg, and proximal humerus is higher in obese compared with normal-weight individuals.^14^^,^^16^^,^^36^ Increased weight affects the biomechanics and direction of falls. Increased weight can be due to an increase in lean mass, fat mass, or both. Greater lean mass is associated with a larger bone size, larger cortical area and thicker cortices, whereas greater fat mass is associated with higher trabecular number and thinner cortices.^40^ The improved bone microarchitecture could be due to heavier mechanical loading with higher body weight or changes in the hormonal milieu (especially estrogen) associated with adiposity that results in lower bone turnover.^20^

It has been suggested that individuals with high BMI are more likely to fall sideways or backwards because of poorer muscle function and postural instability. Furthermore, increased weight generates a greater impact when falling.^16^^,^^17^^,^^41^ In a case–control study of 108 patients ≥40 yr with fracture of the lateral malleolus and 199 fracture-free controls, the prevalence of osteoporosis, osteopenia, and normal aBMD was similar between the two groups.^17^ Having a BMI above 25 kg/m^2^ increased the risk of sustaining an ankle fracture, whereas having osteoporosis did not increase the risk of ankle fractures. Although higher weight is associated with higher BMD and improved bone microarchitecture, this might be insufficient to compensate for other risk factors for fractures in overweight individuals.

The study has several limitations. We lack the tibial lengths, and the ROI used to measure the bone traits was obtained at a standardized distance from the tibial endplate. There are few studies on the errors in bone traits related to differences in the length of long bones using a standardized ROI versus an ROI based on the percentage of the long bone.^42^^,^^43^ As taller women have a standardized ROI located relatively more distally than shorter women, it is likely that the results may be confounded because the bone microarchitecture at the distal tibia is highly variable as a function of tibia length. However, the difference in scan location between a standardized ROI versus percentage-based ROI is generally small when the variation in height is small^44^ as in the current study. Most studies using HR-pQCT for assessment of bone microarchitecture at the distal tibia have used a standardized ROI. Measuring tibia length and using ROI based on the percentage of the tibia length might be a preferable method of determining the association of height and bone traits in future studies.^44^ Another limitation was that including midlife and older women in the analysis does not take into consideration body weight changes since physeal-closure. Results from the ICE FALCON analysis are consistent with weight having a causal effect on cortical CSA, but none of the other bone traits. The results indicate that there are no familial confounders affecting both weight and bone traits, but there could be unmeasured confounders affecting both weight and bone traits. The moderate sample size could have resulted in a lack of statistical power, and the evidence of causation for weight and bone traits might have been too weak to be detected in this study. The relationship between weight and bone fragility is complex, and there are numerous factors affecting the weight, for instance nutrition and diseases such as diabetes mellitus and hypothyroidism,^16^ which might have confounded the results. Further research is warranted to confirm these findings in men. Strengths of this study include the use of HR-pQCT which allowed examination of bone microarchitecture and comparing vBMD with aBMD from DXA scans. Another strength is the use of twin data and the unique ICE FALCON approach, which makes it possible to test consistency with causation in cross-sectional data.

In conclusion, higher stature in women was associated with a weaker cortical bone microarchitecture. In contrast, higher weight was associated with improved cortical and trabecular bone microarchitecture. The attenuation of the cross-pair cross-trait associations after accounting for the within-individual associations was consistent with height having a causal effect on the weaker cortical microarchitecture, whereas weight had a casual effect on the cortical CSA and none of the other bone traits. The current findings suggest that HR-pQCT measurements are useful for exploring the pathophysiology of bone fragility, as well as potential causal pathways for the relationships between height and weight with bone microarchitecture.

## Data Availability

The data used in this study are available from the corresponding author upon reasonable request.
